# Patients with tuberculosis and diabetes show altered clinical and biochemical parameters during anti-TB treatment

**DOI:** 10.1038/s41598-026-36529-8

**Published:** 2026-02-04

**Authors:** Augustine Boadu Asare, Prince Asare, Michelle Yeboah-Manu, Stephen Osei-Wusu, Emelia Danso Konadu, Adwoa Asante-Poku, Yayra Klinogo, Abraham Adjei, Desmond Omane Acheampong, Jane S. Afriyie-Mensah, Atiase Yacoba, Dorothy Yeboah-Manu

**Affiliations:** 1 Department of Bacteriology, Noguchi Memorial Institute for Medical Research–University of Ghana, Accra, Ghana; 2 Department of Biomedical Sciences, School of Allied Health Sciences, University of Cape Coast, Cape Coast, Ghana; 3 University of Ghana Medical Center, University of Ghana, Legon, Ghana; 4 Department of Chest Diseases, Korle-Bu Teaching Hospital, Accra, Ghana; 5 University of Ghana Medical School, Accra, Ghana; 6 National Diabetes Management and Research Center, Korle-Bu Teaching Hospital, Accra, Ghana

**Keywords:** Tuberculosis, Type-2 diabetes mellitus, TB-DM comorbidity, Hyponatremia, Dyslipidemia, Treatment outcomes, Biomarkers, Diseases, Endocrinology, Medical research

## Abstract

Type-2 diabetes mellitus (DM) increases tuberculosis (TB) risk and can worsen treatment outcomes. Both diseases and their treatments induce significant metabolic and biochemical perturbations that influence disease progression and management. This study longitudinally evaluated clinical, metabolic, and serum biochemical changes in patients with pulmonary TB with and without DM before and during anti-TB therapy. Ninety-five adult patients newly diagnosed with pulmonary TB in Ghana were stratified into TB-Only (*n* = 49; HbA1c < 6.5%) and TB-DM (*n* = 46; HbA1c ≥ 6.5%) groups, including treated (TB-DMt) and untreated (TB-DMnt) diabetes subgroups. Serum samples collected at baseline (t_0_), day 28 (t_28_), and day 56 (t_56_) were analyzed for electrolytes, renal function, liver enzymes, and lipid profiles using validated clinical chemistry analyzer. TB-DM cohorts exhibited significantly lower chloride levels at all time points relative to the TB-Only cohort (e.g., 98 vs. 100 mmol/L at t_0_, *p* < 0.001). Hyponatremia (serum sodium < 136 mmol/L) was prevalent during the intensive anti-TB treatment phase, affecting 53.1% of TB-Only patients, 61.1% of TB-DMt patients, and 70.0% of TB-DMnt patients. Liver function tests revealed elevated bilirubin, gamma-glutamyl transferase (g-GT), alkaline phosphatase (ALP), and alanine aminotransferase (ALT) levels, particularly in TB-DMnt patients, with normalization over time. Lipid profiles showed a pro-atherogenic pattern with elevated triglycerides and total cholesterol (*p* < 0.05). High-density and low-density lipoproteins were increased at select time points. Positive correlations were noted among albumin, cholesterol fractions, and electrolytes. Primary microbiological treatment outcomes, including sputum conversion and completion rates, were similar regardless of diabetic status. The distinctive metabolic and biochemical derangements in TB-DM, especially untreated diabetes, highlight the importance of integrated clinical management. Elevated hepatic enzymes in TB-DMnt may delay metformin initiation, suggesting a need to optimize timing post hepatic recovery, while the prevalence of hyponatremia underscores the need for routine electrolyte monitoring in TB patients with diabetes. The dysregulated lipid profile highlights cardiovascular risk that warrants routine monitoring. Despite metabolic challenges, effective TB treatment outcomes are achievable with comprehensive care.

**Supplementary Information:**

The online version contains supplementary material available at 10.1038/s41598-026-36529-8.

## Introduction

Type-2 diabetes mellitus (DM) and tuberculosis (TB) are significant global health challenges impacting millions and burdening health systems worldwide. Currently, an estimated 589 million adults aged 20–79 are living with DM^1^. In Africa, approximately 24 million adults were living with DM in 2021, a figure expected to rise to 55 million by 2045, representing a 129% increase^1,2^. DM is a complex chronic disease characterized by impaired glucose regulation, immune dysfunction, and systemic inflammation, driven by multifactorial aetiologies including genetic predisposition and obesity.

Active TB disease, caused by the members of *Mycobacterium tuberculosis* complex (MTBC), disrupts host metabolic pathways and immune defenses and is associated with poorer outcomes in patients with DM. Systematic reviews and meta-analyses have demonstrated that DM adversely affects TB treatment outcomes, increasing risks of treatment failure, relapse, and mortality^3,4^.

In 2024, an estimated 10.7 million individuals developed TB globally, predominantly in men (54%), with the WHO Africa, South-East Asia, and Western Pacific regions representing nearly 90% of cases^5^. TB cases linked to undernourishment, diabetes, alcohol use, smoking, and HIV infection numbered approximately 971,000, 929,000, 739,000, 696,000, and 570,000, respectively^5^. Both TB and DM, individually and in combination, along with their respective treatments, profoundly affect physiology and metabolism, complicating disease management and increasing the risk of adverse effects including hepatotoxicity, nephrotoxicity, and immune dysregulation, which may delay treatment success^6–10^.

Although timely anti-TB therapy is effective, coexisting DM complicates disease management, requiring integrated healthcare strategies^11^. While substantial research has explored the association between TB and DM comorbidity, the specific biochemical and clinical profile alterations during TB treatment in patients with DM remain insufficiently characterized. Serum biomarker analysis presents valuable opportunities for early detection of medication side effects and infection-related complications, thereby informing timely treatment adjustments. Investigating the biochemical differences associated with DM in patients with TB could enhance monitoring of treatment response, improve diagnostic precision, and optimize patient care^12,13^. This study aims to characterize the biochemical changes in newly diagnosed pulmonary TB patients with and without DM, both prior to and during anti-TB therapy.

## Methods and materials

### Study design and inclusion criteria

**Fig. 1:**
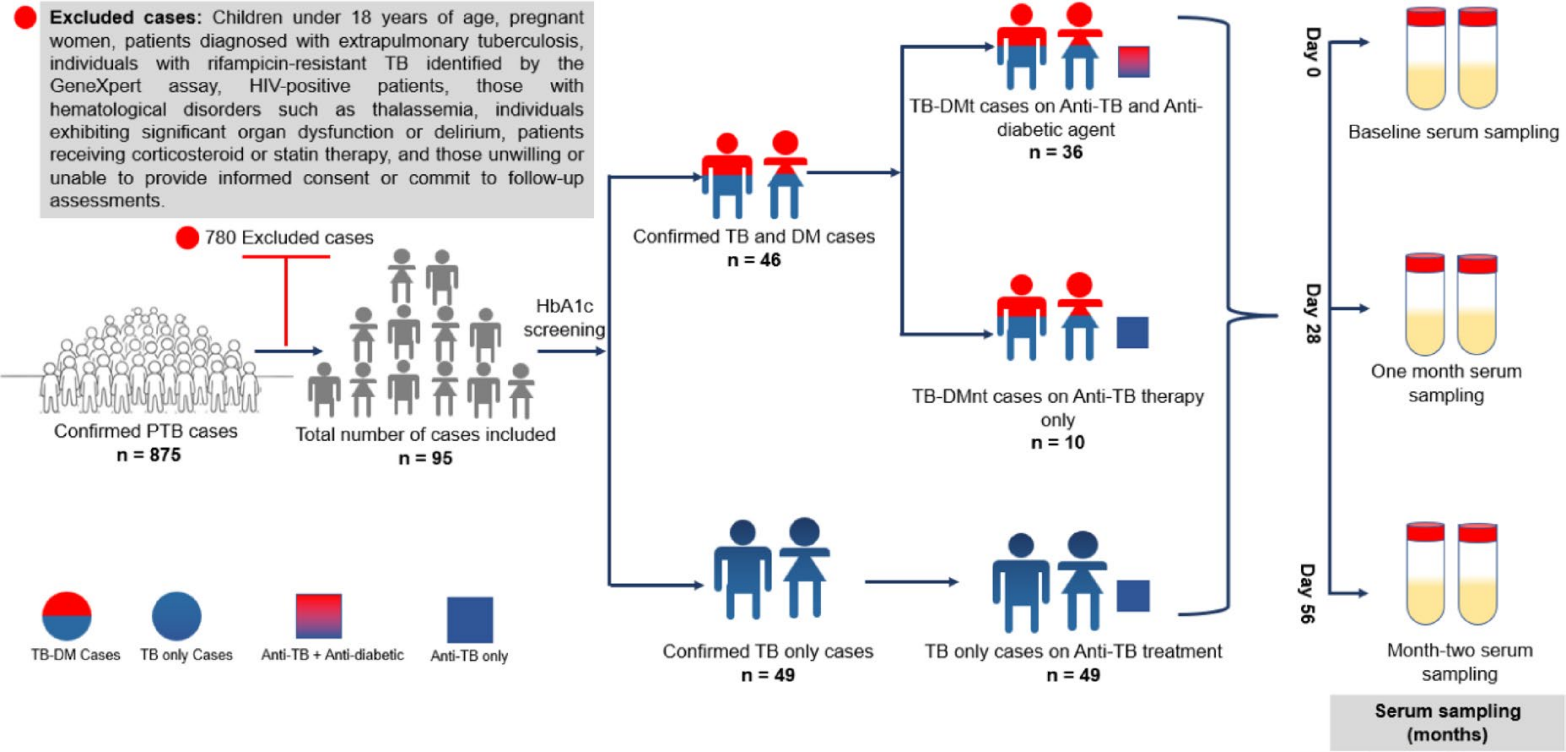
Pipeline from participant recruitment, through series of blood glucose & sera screenings, and monitoring before, and during TB and/or DM treatment. (Prospective study involving 95 newly diagnosed pulmonary TB cases. Categorization into cohorts: TB-Only (HbA1c < 6.5%), TB-DMt (HbA1c ≥ 6.5%; established DM on metformin treatment at baseline), and TB-DMnt (HbA1c ≥ 6.5%; patients diagnosed with dysglycaemia at study enrollment who did not require pharmacological treatment for diabetes). Serial sera assessments at multiple time points during TB treatment.

This was a hospital-based prospective longitudinal cohort study conducted at the Department of Chest Diseases, Korle-Bu Teaching Hospital (KBTH), Ghana, over two years (October 20, 2020 to November 30, 2023). Adults aged 18 years and older, newly diagnosed with drug-sensitive pulmonary tuberculosis (PTB), were enrolled. Diagnosis was primarily confirmed using the Xpert MTB/RIF Ultra assay (Cepheid, USA), which offers enhanced sensitivity by targeting multicopy DNA sequences (*IS6110* and *IS1081*). ‘Trace’ results were interpreted as MTBC positive with indeterminate rifampicin resistance, in line with WHO guidelines; clinical assessment and additional tests informed rifampicin susceptibility determination. Chest radiography served as a secondary confirmatory tool. Patients were excluded if they had rifampicin-resistant tuberculosis detected by the Ultra assay, HIV co-infection, extrapulmonary TB, pregnancy, ongoing corticosteroid or statin therapy, haematological disorders such as thalassemia, active delirium, or any significant organ dysfunction (Fig. 1). All participants provided written informed consent and agreed to follow study procedures and scheduled follow-ups.

### Sample size and outcome measures

The final sample size of 95 participants was determined based on preliminary data and existing literature indicating detectable biochemical differences between TB-Only and TB-DM cohorts^12,13^. The primary outcome was the longitudinal change in serum biochemical parameters among patients with TB, stratified by diabetes status. Secondary outcomes included the incidence of treatment-emergent adverse events (TEAEs), sputum conversion rates during therapy, and clinical outcomes defined by WHO criteria (cured, treatment completed, deceased, or lost to follow-up)^14^.

### Study cohorts and specimen collection

Confirmed PTB cases were categorized into two cohorts based on glycated haemoglobin (HbA1c) levels per the American Diabetes Association 2020 guidelines (10.2337/dc22-SINT). Cohorts were defined as TB-Only (HbA1c < 6.5%) and comorbid TB-DM (HbA1c ≥ 6.5%) as described by Ugarte-Gil et al.^11.^ The TB-DM group was further stratified into TB-DMt (known DM on metformin therapy at baseline) and TB-DMnt (patients diagnosed with dysglycaemia at study enrollment who did not require pharmacological treatment for diabetes) classified according to the treating physician’s clinical judgement supported by blood glucose monitoring. Initial diabetes screening used fasting blood glucose (FBG) ≥ 7 mmol/L, confirmed by HbA1c testing.

Peripheral blood (8 mL) was collected via brachial venipuncture into serum separator tubes between 08:00–10:00 following an 8-hour fast. Samples were processed by in-house phlebotomists. Serum was obtained through centrifugation at 3,000 rpm for 15 min, aliquoted into sterile microtubes, and analyzed using the Beckman Coulter DxC 700 AU clinical chemistry analyzer for panels including urea, creatinine, electrolytes (potassium, chloride, sodium, bicarbonate), liver enzymes (AST, ALT), bilirubin, and lipid profile (triglycerides, HDL, LDL, total cholesterol) with related ratios.

Serial serum samples for biochemical panels were collected at baseline (t_0_), day 28 (t_28_), and day 56 (t_56_) during the intensive phase of anti-TB therapy (Fig. 1).

Microbiological assessments were carried out during every study visit in tandem with the serum sampling schedule, utilizing early morning sputum samples from the patients. The sputum specimens were analyzed for the detection of acid-fast bacilli (AFB) and cultured on Lowenstein-Jensen (LJ) media, following the methodology outlined by Yeboah-Manu et al.^15^

### Regimen

This was a non-interventional observational study. Patients received the standard anti-TB regimen consisting of a fixed-dose combination (FDC) of isoniazid (H), rifampin (R), pyrazinamide (Z), and ethambutol (E) daily during the initial two-month intensive phase, followed by HR daily for 4 months, aligned with WHO guidelines^16^. Patients in the TB-DMt group concurrently received metformin monotherapy (500-1000 mg BID daily; median 1500 mg/day) as prescribed by their physicians. To prevent isoniazid-associated peripheral neuropathy, all patients were prescribed 10 mg of pyridoxine once daily^16^.

### Laboratory investigation of serum samples

Sera were separated from whole blood samples by centrifugation at 3,000 rpm for 15 min and aliquoted into sterile polypropylene microtubes. The serum samples were then analyzed using the Beckman Coulter DxC 700 AU clinical chemistry analyzer.

Serum parameters evaluated included urea (UREA), creatinine (CTN), electrolytes (potassium [K^+^], chloride [Cl^−^], sodium [Na^+^], total bicarbonate [HCO₃⁻]), liver enzymes—aspartate aminotransferase (AST) and alanine aminotransferase (ALT), total and direct bilirubin, as well as lipid profile components including triglycerides (TG), high-density lipoprotein (HDL), low-density lipoprotein (LDL), total cholesterol (TC), and related ratios.

### Data analysis

This was performed using Stata version 14.2 (StataCorp LLC, College Station, TX, USA) for comprehensive statistical computations and GraphPad Prism 8.4.0 for data visualization and supplementary analyses. Initial data management, cleaning, and validation were conducted in Microsoft Access. Categorical variables were summarized as frequencies and percentages accompanied by 95% confidence intervals. Continuous variables were assessed for normality within each group using the Shapiro-Wilk test. Non-normally distributed continuous data were reported as medians with interquartile ranges (IQR). Group comparisons for continuous variables involved non-parametric methods: Mann-Whitney U test (two-sample Wilcoxon rank-sum test), for pairwise comparisons between groups. For categorical variables, Pearson’s chi-square test was applied unless expected cell counts were less than five, in which case Fisher’s exact test was used. Statistical significance was evaluated using two-tailed p-values, with *p* < 0.05 considered significant.

### Ethics statement

Ethical approval for this study protocol was obtained from both the Scientific Committee and the Institutional Review Board (IRB) of the Noguchi Memorial Institute for Medical Research (NMIMR), University of Ghana (Federal wide assurance number: FWA00001824), and the Ethics Review Committee of the Korle-Bu Teaching Hospital (KBTH-IRB/00081/2020). Furthermore, this study was conducted in accordance with the principles outlined in the Declaration of Helsinki (version 2013). Prior to any data collection, written informed consent was obtained from literate participants, while consent was secured from the legal guardians of all illiterate participants. All methods and procedures adhered to established ethical standards and regulatory requirements.

### Role of the funding source

The funders had no role in the study design, collection, analysis and interpretation of data, manuscript writing, or decision to publish.

## Results

### Patient characteristics

Out of 875 newly diagnosed pulmonary tuberculosis (PTB) patients screened, 95 were deemed eligible and consented for inclusion in this study (Fig. 1). The cohort included 49 patients with TB-Only and 46 with comorbid TB-DM. The TB-DM group was significantly older, with a median age of 55 years (IQR 48–58), compared to 28 years (IQR 24–36) in the TB-Only group (*p* < 0.001). Median weight was higher in the TB-DM group at 60 kg (IQR 52–70) versus 55 kg (IQR 50–60) in TB-Only (*p* = 0.04). Similarly, median BMI was elevated in TB-DM participants at 21 kg/m² (IQR 19–27) compared to 20 kg/m² (IQR 18–21) in TB-Only (*p* = 0.01) (Table 1).

Females accounted for 28.6% (95% CI 16.1–43.5) of TB-Only and 45.6% (95% CI 31.2–60.5) of TB-DM participants, with no significant gender difference (*p* = 0.09). Marital status differed between groups; 61.2% of TB-Only patients were single compared to 13.0% in TB-DM, while many TB-DM patients were married (71.7%) versus 36.7% of TB-Only (*p* < 0.001). Education level also varied significantly (*p* < 0.001), with higher proportions of TB-DM patients reporting no or primary education. There were no significant differences between groups regarding occupation, smoking history, or alcohol abuse (*P* > 0.05).

**Table 1:** Sociodemographic data of participants (*N* = 95).

Variable	TB-Only (*n*=49) Median (IQR)	TB-DM (*n*=46) Median (IQR)	*p*-value
Age (years)	28 (24–36)	55 (48–58)	<0.0001
Weight (kg)	55 (50–60)	60 (52–70)	0.043
BMI (kg/m^2^)	20 (18–21)	21 (19–27)	0.009
	**Count (%**,** 95% CI)**	**Count (%**,** 95% CI)**	
Gender			0.094
Female	14 (28.6%, 16.1–43.5)	21 (45.6%, 31.2–60.5)	
Male	35 (71.4%, 56.5–83.9)	25 (54.4%, 39.5–68.8)	
Marital status			<0.0001
Single	30 (61.2%, 46.2–74.8)	6 (13.0%, 5.0–26.9)	
Married	18 (36.7%, 23.1–51.6)	33 (71.7%, 56.5–83.5)	
Divorce	1 (2.1%, 0.1–11.4)	5 (10.9%, 4.3–23.5)	
Widowed	0 (0.0%, 0.0–7.4)	2 (4.4%, 0.5–15.0)	
Education			0.002
None	3 (6.1%, 1.3–16.9)	8 (17.4%, 8.0–31.7)	
Primary	4 (8.2%, 2.3–19.6)	8 (17.4%, 8.0–31.7)	
Middle/JHS	13 (26.5%, 15.0–41.2)	21 (45.7%, 31.2–60.5)	
Secondary	20 (40.8%, 27.0–55.8)	5 (10.9%, 4.3–23.5)	
Tertiary	9 (18.4%, 9.0–32.1)	4 (8.7%, 2.4–20.8)	
Occupation (*n*=89)			0.113
Unemployed	9 (19.2%, 9.1–33.3)	4 (9.5%, 2.7–22.6)	
Unskilled labour	22 (46.8%, 32.1–61.9)	29 (69.1%, 52.9–82.4)	
Skilled labour	16 (34.0%, 20.4–49.9)	9 (21.4%, 10.2–36.8)	
Hx of smoking			0.597
No	39 (79.6%, 65.7–89.8)	39 (84.8%, 71.7–93.1)	
Yes	10 (20.4%, 10.2–34.3)	7 (15.2%, 6.9–28.3)	
Hx of alcohol abuse			0.407
No	17 (34.7%, 22.0–49.4)	20 (43.5%, 29.2–58.8)	
Yes	32 (65.3%, 50.6–78.0)	26 (56.5%, 41.2–70.8)	
Hx of previous TB treatment and outcome			0.113
New case	49 (100%, 92.6–100)	43 (93.5%, 81.5–98.6)	
Relapse	0 (0.0%, 0.0–7.4)	3 (6.5%, 1.4–17.8)	

Data are presented as median (interquartile range) for continuous variables and count (percentage, 95% confidence interval) for categorical variables. P-values were obtained using Mann-Whitney U tests for continuous variables and Pearson’s chi-square or Fisher’s exact tests for categorical variables, as appropriate; BMI, body mass index, Hx (history).

### Clinical presentation pre- and post-intensive phase anti-TB therapy

At baseline, the median fasting blood glucose (FBG) was significantly elevated in the TB-DM cohort at 13.4 mmol/L (IQR 9.2–19.6) compared to 6.6 mmol/L (IQR 5.6–7.9) in the TB-Only group (*p* < 0.001). Similarly, glycated hemoglobin (HbA1c) was higher among TB-DM patients with a median of 8.7% (95% CI 6.8–11.3) versus 5.6% (IQR 5.4–5.9) in TB-only (*p* < 0.001) (Table 2).

The nature of sputum did not significantly differ between groups (*p* = 0.29), with mucopurulent sputum most common. Microscopy positivity grades were comparable (*p* = 0.98), but culture positivity was significantly higher in TB-Only (89.8%) than TB-DM (73.9%, *p* = 0.04). Chest radiograph abnormalities (e.g., lobar infiltrations and cavitations) and symptom profiles were similar, though fever trended more frequent in TB-DM (*p* = 0.05). MTB RIF load categories were also comparable between groups (*p* = 0.67).

Regarding blood pressure, all TB-Only patients were normotensive, whereas 33.3% of TB-DM patients were hypertensive (*p* < 0.001). Symptoms such as extreme and mild cough, night sweats, and chest pains were comparable (*p* > 0.05). Dyspnea was more common in TB-DM (60.9% vs. 44.9%, *p* = 0.15), but not statistically significant. Hemoptysis and fever showed trends toward higher frequency in TB-DM (*p* = 0.05).

At 2-, 3-, and 6-month follow-up (status after the intensive phase; Table 3), TB-DM patients continued to exhibit significantly higher median FBG and HbA1c levels compared to TB-Only (*p* < 0.001 for all time points). Sputum nature at 2 months indicated significant differences (*p* < 0.001), with more mucopurulent sputum in TB-DM.

Microscopy positivity and culture positivity rates at 2 months were not significantly different (*p* = 0.37 and 0.72, respectively). Dyspnea remained more prevalent in TB-DM at 2 months (*p* = 0.01).

By 6 months, treatment outcomes—cured, completed, dead, lost to follow-up, and defaulters—did not differ significantly between cohorts (*p* = 0.42). Treatment emergent adverse events (TEAEs) were more frequent in TB-DM but lacked statistical significance (Table 3).

**Table 2:** Baseline clinical presentation by cohorts (*N* = 95).

Variable	TB-Only (*n*=49) Median (IQR)	TB-DM (*n*=46) Median (IQR)	*p*-value
Fasting blood glucose (FBG mmol/L, *N*=95)	6.6 (5.6–7.9), *n*=49	13.4 (9.2–19.6), *n*=46	<0.0001
Glycated haemoglobin (HbA1c %, *N*=70)	5.6 (5.4–5.9), *n*=25	8.7 (6.8–11.3), *n*=45	<0.0001
	**Count (%**,** 95% CI)**	**Count (%**,** 95% CI)**	
Nature of sputum n (%)			0.285
Blood stained	3 (6.1%, 1.3–16.9)	2 (4.4%, 0.5–15.0)	
Mucopurulent	24 (49.0%, 34.8–63.3)	30 (65.2%, 50.0–78.3)	
Mucosalivary	15 (30.6%, 18.1–45.4)	7 (15.2%, 6.2–28.6)	
Salivary	7 (14.3%, 6.0–27.9)	7 (15.2%, 6.2–28.6)	
MTB RIF load			
MTB RIF-high	8 (16.3%, 7.3–29.7)	10 (21.7%, 11.0–36.4)	0.668
MTB RIF-med	27 (55.1%, 40.2–69.3)	26 (56.6%, 41.2–70.7)	
MTB RIF-low	14 (28.6%, 16.4–43.6)	10 (21.7%, 11.0–36.4)	
Microscopy degree of positivity n (%)			
+	22 (61.1%, 45.5–75.0)	18 (56.3%, 41.2–70.7)	0.978
++	6 (16.7%, 6.4–32.8)	8 (25.0%, 13.6–40.2)	
+++	6 (16.7%, 6.4–32.8)	6 (18.8%, 8.0–35.2)	
Scanty	2 (5.6%, 0.7–18.7)	0 (0.0%, 0.0–7.9)	
Culture positivity n (%)			
Negative	5 (10.2%, 3.4–22.2)	12 (26.1%, 14.2–41.1)	0.044
Positive	44 (89.8%, 77.8–96.6)	34 (73.9%, 58.9–85.8)	
Blood pressure n (%)			
Normal	48 (100%, 92.6–100)	30 (66.7%, 51.8–79.9)	<0.0001
Hypertensive	0 (0%, 0.0–7.4)	15 (33.3%, 20.1–48.1)	
**Chest radiograph**			
a. No. of patients with abnormal chest X-ray (N=58)	TB-Only (n = 29)	TB-DM (n = 29)	
b. Infiltrations			
Lower lobe			
No	11 (37.9%, 20.7–57.7)	10 (34.5%, 18.6–53.2)	1.000
Yes	18 (62.1%, 42.3–79.3)	19 (65.5%, 46.8–81.4)	
Middle lobe			
No	5 (17.2%, 5.8–35.8)	4 (13.8%, 3.8–31.7)	1.000
Yes	24 (82.8%, 64.2–94.2)	25 (86.2%, 68.3–96.1)	
Upper lobe			
No	4 (13.8%, 3.8–31.7)	10 (34.5%, 18.6–53.2)	0.123
Yes	25 (86.2%, 68.3–96.1)	19 (65.5%, 46.8–81.4)	
c. Cavitation			
Lower lobe			
No	27 (93.1%, 77.2–99.2)	25 (86.2%, 68.3–96.1)	0.670
Yes	2 (6.9%, 0.8–22.8)	4 (13.8%, 3.8–31.7)	
Middle lobe			
No	22 (75.9%, 56.5–89.7)	21 (72.4%, 53.3–87.3)	1.000
Yes	7 (24.1%, 10.3–43.5)	8 (27.6%, 12.7–46.7)	
Upper lobe			
No	17 (58.6%, 40.7–75.5)	20 (69.0%, 50.4–83.5)	0.585
Yes	12 (41.4%, 24.5–59.3)	9 (31.0%, 16.5–49.6)	
Symptoms (*N*=95)			
Extreme cough	38 (77.6%, 63.6–88.0)	39 (84.8%, 71.1–93.7)	0.438
Mild cough	9 (18.4%, 9.2–31.4)	7 (15.2%, 6.6–28.8)	0.787
Night sweats	34 (69.4%, 54.6–81.7)	31 (67.4%, 52.2–80.4)	1.000
Chest pains	38 (77.6%, 63.6–88.0)	34 (73.9%, 59.3–85.7)	0.811
Dyspnea	22 (44.9%, 31.1–59.5)	28 (60.9%, 46.2–74.0)	0.151
Hemoptysis	4 (8.2%, 2.3–19.6)	10 (21.7%, 11.3–36.3)	0.084
Fever	8 (16.3%, 7.4–30.5)	16 (34.8%, 22.1–49.7)	0.058
Weight loss	48 (98.0%, 89.3–99.9)	42 (91.3%, 79.4–97.6)	0.195

Sample sizes: TB-Only group (*n* = 49) and TB-DM group (*n* = 46), except where otherwise indicated. P-values are from Mann-Whitney U tests for continuous data and Pearson’s chi-square or Fisher’s exact test for categorical data, as appropriate; Blood pressure: Normal (systole 100 < x < 130; diastole 60 < x < 80 mmHg); Hypertensive (x > 130; diastole x > 80 mmHg). Mild cough, 3–4 times of cough per 24-hr with no mucus; Extreme cough, > 4 times cough per 24-hr with purulent discharged, difficult in swallowing and lasting more than 2-week.

**Table 3:** Clinical presentation by cohorts at 2-, 3-, and 6-month follow-up: status after the intensive phase of anti-TB treatment.

Variable	TB-Only (*n*=49) Median (IQR)	TB-DM (*n*=46) Median (IQR)	*P* value
Fasting blood glucose (FBG mmol/L)			
At 3-month (N=64)	6.1 (5.7–6.9), *n*=23	12.9 (7.4–16.7), *n*=41	<0.0001
At 6-month (N=64)	6.0 (5.0-6.6), *n*=23	11.0 (7.0-16), *n*=41	<0.0001
Glycated haemoglobin (HbA1c %)			
At 3-month (N=63)	5.3 (4.8–5.9), *n*=23	8.1 (6.9–9.5), *n*=40	<0.0001
At 6-month (N=63)	5.4 (5.1-5.7), *n*=23	7.7 (5.8–9.3), *n*=40	<0.0001
	**Count (%**,** 95% CI)**	**Count (%**,** 95% CI)**	
Nature of sputum at 2-month (N = 88)			
Blood stained	0 (0.0%, 0.0–7.2)	1 (2.5%, 0.1–13.2)	0.001
Mucopurulent	10 (20.8%, 11.1–34.5)	21 (52.5%, 37.5–67.0)	
Mucosalivary	20 (41.7%, 28.2–56.7)	14 (35.0%, 22.1–50.5)	
Salivary	18 (37.5%, 24.0–52.7)	4 (10.0%, 3.3–23.6)	
Microscopy positivity (*N* = 95)			
Negative	37 (75.1%, 59.7–84.4)	30 (65.2%, 45.9–75.1)	0.368
Positive (Scanty, +, ++, +++)	12 (24.9%, 15.6–40.3)	16 (34.8%, 24.9–54.1)	
Culture positivity at 2 months (*N* = 89)			
Negativity	39 (79.6%, 65.0–89.5)	29 (72.5%, 57.0–84.8)	0.716
Positive	4 (8.2%, 2.3–20.0)	5 (12.5%, 4.5–26.9)	
**Under observation	6 (12.2%, 4.7–25.6)	6 (15.0%, 5.7–29.8)	
**Symptoms **(***N*** ***= 94***)	**TB-Only (n=48)**	**TB-DM (n=46)**	
Extreme cough	7 (14.6%, 6.0–27.5)	7 (15.2%, 6.4–29.1)	1.000
Mild cough	32 (66.7%, 52.4–78.9)	36 (78.3%, 64.1–88.6)	0.258
Chest pains	15 (31.3%, 19.1–45.9)	21 (45.7%, 31.2–60.7)	0.145
Dyspnea	5 (10.4%, 3.5–22.7)	15 (32.6%, 19.5–47.9)	0.011
Hemoptysis	0 (0.0%, 0.0–7.5)	1 (2.2%, 0.1–12.0)	0.484
Fever	1 (2.1%, 0.1–11.3)	2 (4.4%, 0.5–14.8)	0.609
Weight loss	2 (4.2%, 0.5–14.3)	4 (8.7%, 2.4–20.8)	0.426
TEAEs	3 (6.3%, 1.3–17.3)	7 (15.2%, 6.4–29.2)	0.156
Treatment outcomes at 6 months (*N*=95)			0.418
Cured	35 (71.4%, 57.8–83.4)	23 (50.0%, 35.5–64.5)	
Completed	4 (8.2%, 2.3–19.6)	6 (13.0%, 5.1–26.8)	
Dead	1 (2.0%, 0.1–10.9)	4 (8.7%, 2.4–20.8)	
^#^Lost to follow up	1 (2.0%, 0.1–10.9)	0 (0.0%, 0.0–7.9)	
^#^Defaulters	8 (16.3%, 8.0–29.1)	13 (28.3%, 17.1–42.7)	

Sample sizes: TB-Only group (*n* = 49) and TB-DM group (*n* = 46), except where otherwise indicated. P-values are from Mann-Whitney U tests for continuous data and Pearson’s chi-square or Fisher’s exact test for categorical data, as appropriate; Intensive phase was the first 2 months of treatment. Rx, treatment; TEAEs, treatment emergent adverse effects (vomiting, peripheral neuropathy, headache, etc.). Treatment outcome based on WHO standards. ^#^lost to follow-up as a treatment outcome for patients who stopped treatment for at least two months, while “defaulters” refer to patients excluded from analysis due to unavailable or incomplete follow-up data. **collected samples undergoing incubation and monitoring to detect the growth of *Mycobacterium tuberculosis*.

### Serum biochemical changes in tuberculosis and diabetes comorbidity patient cohorts before and during treatment

**Fig. 2:**
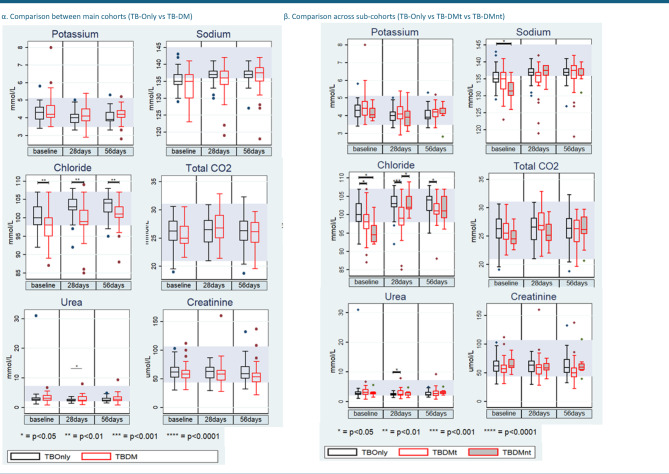
Changes in serum electrolytes and renal panels [(α) main cohorts: TB-Only vs. TB-DM; (β) sub-cohorts: TB-Only vs. TB-DMt vs. TB-DMnt] before and during anti-TB and/or diabetes treatment at the intensive phase. In all box plots, the horizontal line represents the median, and the box depicts the interquartile range (IQR, 25th to 75th percentile). P-values represent the results of pairwise comparisons between the three cohorts (TB-Only, TB-DMt, TB-DMnt) using two-sample Wilcoxon rank‐sum (Mann–Whitney) test.

Serum electrolyte levels of potassium (K⁺) and total bicarbonate (HCO₃⁻) remained consistent across all cohorts and time points (*p* > 0.05) (Fig. 2; Supplementary Tables 1–3).

Chloride (Cl⁻) levels were significantly lower in diabetic cohorts across all time points. At baseline, median chloride was 100 mmol/L (IQR 98–103) in TB-Only compared to 98 mmol/L (96–100) in TB-DMt (*p* = 0.03) and 95 mmol/L (93–97) in TB-DMnt (*p* = 0.001). This difference remained significant at day 28 and day 56 (*p* < 0.05).

Sodium (Na⁺) levels were significantly lower in the TB-DMnt group compared to TB-Only at baseline (*p* = 0.02), with median values of 132 mmol/L (IQR 130–134) versus 135 mmol/L (IQR 134–137), respectively; TB-DMt patients had intermediate median sodium of 135 mmol/L (IQR 130–137). Sodium concentrations remained stable over time, showing no significant differences between groups at days 28 and 56.

Renal function markers including urea, creatinine, and estimated glomerular filtration rate (eGFR) were comparable among TB-Only, TB-DMt, and TB-DMnt cohorts at baseline (all *p* > 0.05). However, by day 28, eGFR was significantly reduced in both TB-DM (*p* = 0.04) and TB-DMt (*p* = 0.02) groups relative to TB-Only. Creatinine at day 28 showed a trend toward being lower in TB-DMt compared to TB-Only (*p* = 0.07). At day 56, creatinine levels were significantly elevated in the TB-Only group compared with both TB-DM (*p* = 0.02) and TB-DMt (*p* = 0.01) cohorts.

**Fig. 3:**
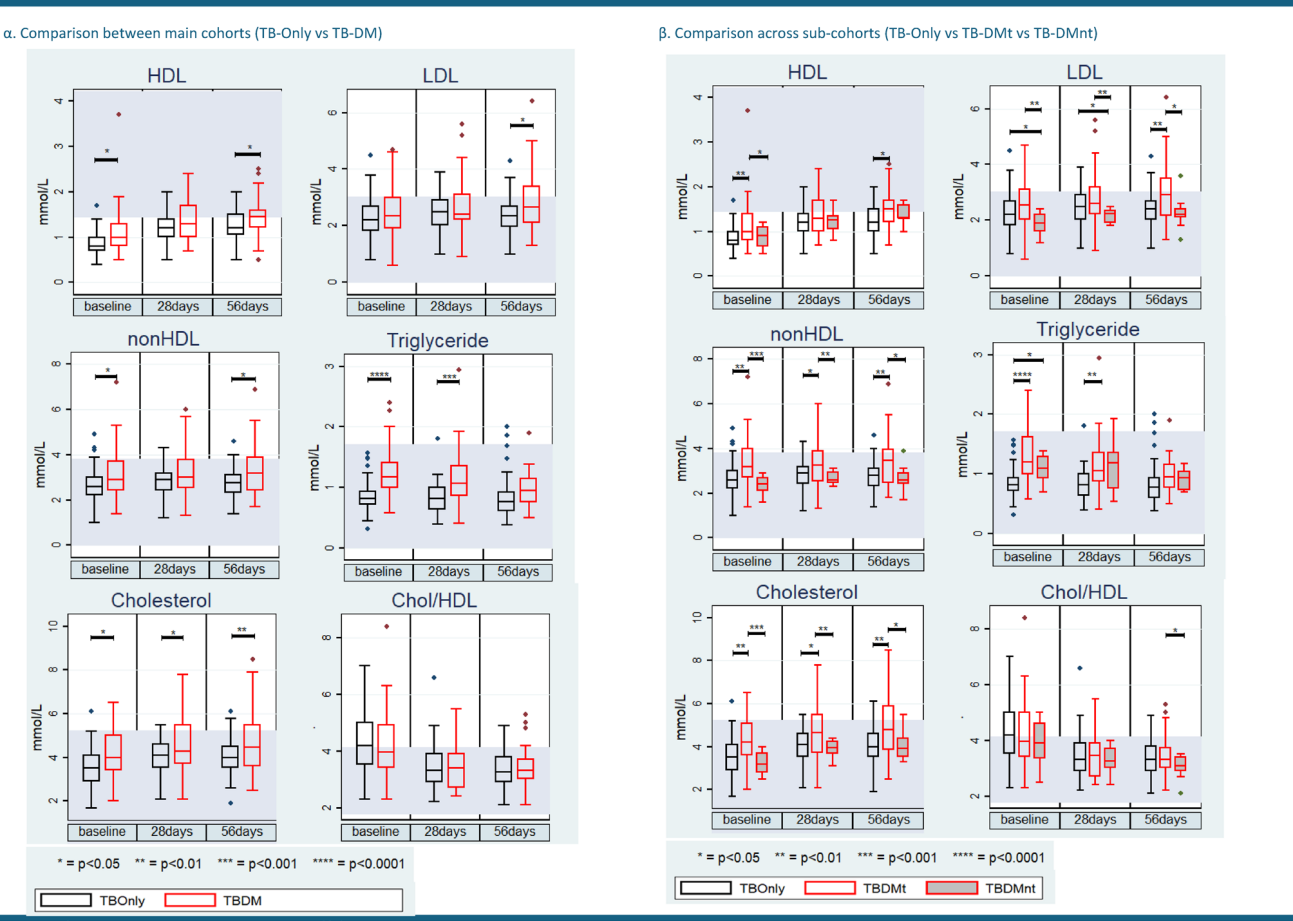
Changes in serum lipid panels [(α) main cohorts and (β) sub-cohorts as above] over the treatment period. In all box plots, the horizontal line represents the median, and the box depicts the interquartile range (IQR, 25th to 75th percentile). P-values represent the results of pairwise comparisons between the three cohorts (TB-Only, TB-DMt, TB-DMnt) using two-sample Wilcoxon rank‐sum (Mann–Whitney) test.

TB-DMt patients consistently exhibited elevated total cholesterol and triglyceride levels compared to TB-Only cohort across all time points (*p* < 0.05). HDL cholesterol was significantly higher in the TB-DMt group at baseline (*p* < 0.001) and day 56 (*p* = 0.03), while LDL cholesterol significantly increased in TB-DMt at day 56 versus TB-Only (*p* < 0.01). Conversely, the TB-DMnt cohort showed significantly lower total cholesterol and LDL cholesterol at baseline (*p* = 0.01 and *p* = 0.02, respectively) and maintained lower LDL levels at follow-up days 28 (*p* = 0.04) and 56 (*p* = 0.03) compared to TB-DMt. Lipid levels in the TB-DMnt cohort were generally comparable to TB-Only across all observations (Fig. 3; Supplementary Tables 1–3).

**Fig. 4:**
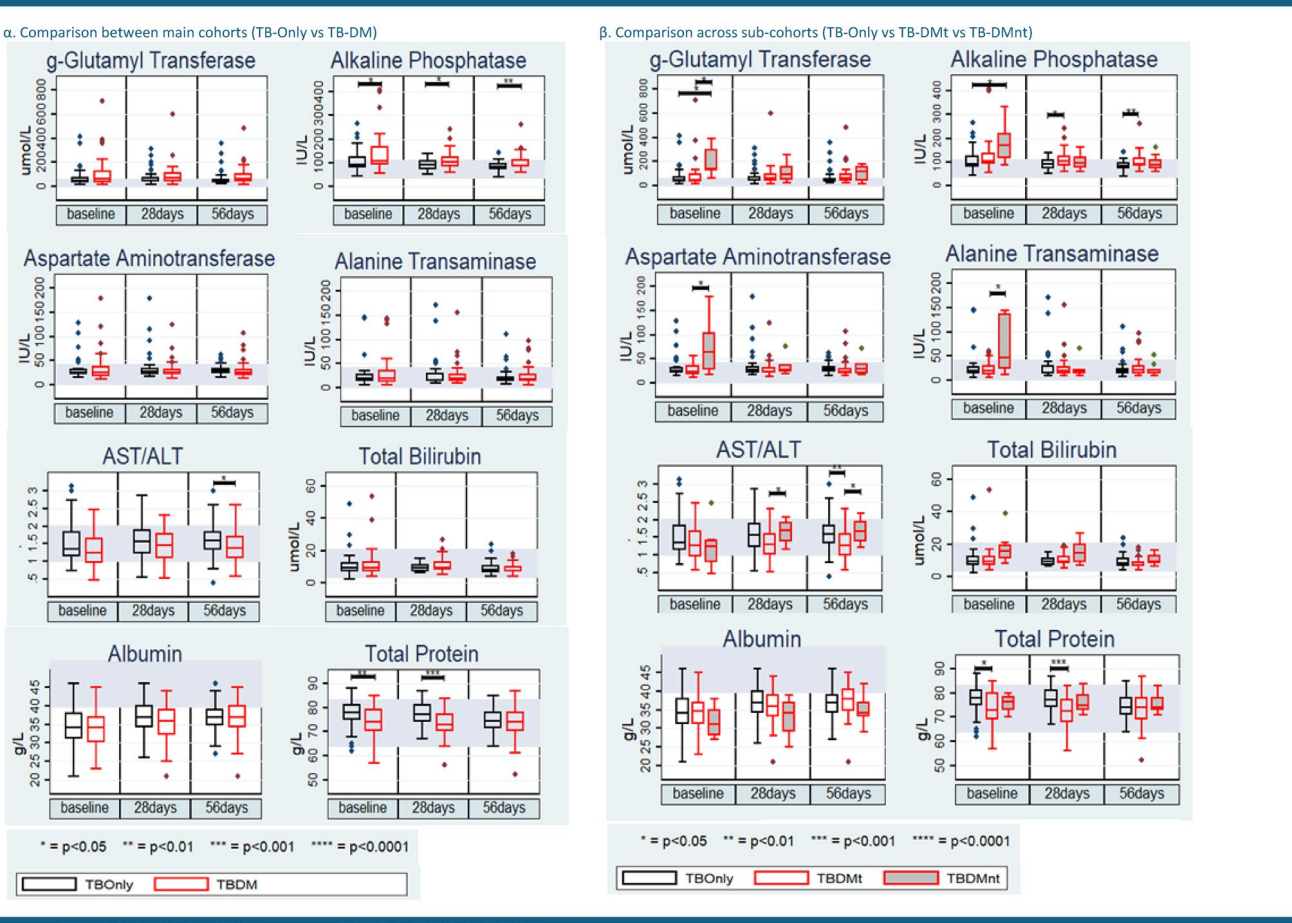
Changes in serum liver function markers [(α) main cohorts and (β) sub-cohorts as above] over time. In all box plots, the horizontal line represents the median, and the box depicts the interquartile range (IQR, 25th to 75th percentile). P-values represent the results of pairwise comparisons between the three cohorts (TB-Only, TB-DMt, TB-DMnt) using two-sample Wilcoxon rank‐sum (Mann–Whitney) test.

Serum bilirubin (total *p* = 0.01, conjugated *p* = 0.01), Gamma-glutamyl transferase (g-GT) (*p* < 0.001), and Alkaline phosphatase (ALP) (*p* < 0.001) were significantly higher in TB-DMnt compared to TB-Only at baseline, with total bilirubin remaining consistently higher at day 28 (*p* < 0.05). g-GT levels were markedly increased at baseline in TB-DMnt (*p* < 0.001) and remained elevated at day 56 (*p* = 0.03). Albumin levels were also significantly higher in the TB-DMnt group at day 56 (*p* = 0.04). Conversely, total serum protein was significantly reduced in TB-DMt patients at baseline (*p* = 0.01) and day 28 (*p* < 0.001) relative to TB-Only. ALP was significantly elevated in the TB-DM group at baseline (*p* = 0.01) and day 28 (*p* < 0.001), but these differences resolved by day 56 (*p* > 0.05) (Fig. 4; Supplementary Tables 1–3).

### Serum biochemical abnormalities in tuberculosis patients with and without diabetes during the intensive phase of anti-TB treatment

Serum biochemical abnormalities were prevalent in TB patients with and without diabetes throughout the intensive phase of anti-TB treatment. At baseline (Day 0, *N* = 95) (Table 4), hyponatremia (sodium < 136 mmol/L) affected over half the patients across groups, with TB-DMnt patients exhibiting the highest prevalence at 70.0% (95% CI, 34.8–93.3). Hyperkalemia (potassium > 5.1 mmol/L) was notably higher in TB-DMt (22.2%, 9.9–39.6) and TB-DMnt (20.0%, 2.5–55.6) compared to TB-Only patients (2.0%, 0.1–10.5). Renal abnormalities, including elevated urea and creatinine and reduced eGFR, were consistently more frequent in the TB-DMt cohort. Liver enzyme elevations (ALT, AST) were comparable among groups, though TB-DMnt patients showed higher proportions. The AST/ALT ratio differed across groups, with TB-DMt patients showing higher prevalence of low ratios (< 1) and TB-Only patients more frequently exhibiting high ratios (> 2). Hypoalbuminemia was most common in TB-Only patients (53.1%, 39.3–66.6). Lipid abnormalities included high prevalence of low HDL cholesterol in all groups, with elevated LDL and total cholesterol more common in TB-DMt patients.

At one month (Day 28) (Table 5), electrolyte and renal abnormalities persisted, especially in TB-DMt patients, with hyperkalemia present in 12.1% (3.3–29.7) of TB-DMt but absent in TB-Only. Liver panel abnormalities followed similar trends. By two months (Day 56) (Table 6), many biochemical abnormalities remained stable, notably hypoalbuminemia in TB-DMnt patients (60.0%, 26.2–87.8). Electrolyte imbalances and lipid panel abnormalities also persisted, underscoring the metabolic challenges faced by TB patients with diabetes during treatment.

**Table 4:** Abnormalities in serum biochemical parameters observed across cohorts, before the initiation of anti-TB treatment (Day 0, *N* = 95).

Parameter	Serum abnormalities	TB-Only (*n* = 49) Count (%, 95% CI)	TB-DMt (*n* = 36) Count (%, 95% CI)	TB-DMnt (*n* = 10) Count (%, 95% CI)
Serum Electrolyte	Hypokalemia (< 3.5 mmol/L)	1 (2.0%, 0.1–10.5)	0 (0.0%, 0.0–7.2)	0 (0.0%, 0.0–25.9)
	Hyperkalemia (> 5.1 mmol/L)	1 (2.0%, 0.1–10.5)	8 (22.2%, 9.9–39.6)	2 (20.0%, 2.5–55.6)
	Low Sodium (< 136 mmol/L)	26 (53.1%, 39.3–66.6)	22 (61.1%, 44.2–76.7)	7 (70.0%, 34.8–93.3)
	Low chloride (< 98 mmol/L)	12 (24.5%, 13.4–38.8)	12 (33.3%, 19.6–49.6)	7 (70.0%, 34.8–93.3)
Renal panels	High urea (> 7.1 mmol/L)	1 (2.0%, 0.1–10.5)	3 (8.3%, 1.8–22.5)	2 (20.0%, 2.5–55.6)
	High creatinine (M: >106 µmol/L; F: >80 µmol/L)	4 (8.2%, 2.3–19.6)	8 (22.2%, 9.9–39.6)	2 (20.0%, 2.5–55.6)
	Low eGFR (18-40years < 89: >40years < 60 mL/min/1.73m^2Y^)	2 (4.1%, 0.5–14.0)	4 (11.1%, 3.1–26.1)	1 (10.0%, 0.3–44.5)
Liver panels	High ALT (M: >41 IU/L; F: >33 IU/L)	4 (8.2%, 2.3–19.6)	5 (13.9%, 4.6–30.7)	5 (50.0%, 18.7–81.3)
	High AST (M: >40 IU/L; F: >32 IU/L)	9 (18.4%, 8.8–32.0)	6 (16.7%, 6.4–34.5)	5 (50.0%, 18.7–81.3)
	Low AST/ALT ratio (< 1)	7 (14.3%, 6.0–27.5)	9 (25.0%, 12.1–43.8)	2 (20.0%, 2.5–55.6)
	High AST/ALT ratio (> 2)	8 (16.3%, 7.3–30.9)	6 (16.7%, 6.4–34.5)	1 (10.0%, 0.3–44.5)
	High ALP (> 105 IU/L)	16 (32.7%, 20.0–47.6)	17 (47.2%, 30.4–64.5)	6 (60.0%, 26.2–87.8)
	High bilirubin (total) (> 20.5 µmol/L)	4 (8.2%, 2.3–19.6)	1 (2.8%, 0.1–14.5)	2 (20.0%, 2.5–55.6)
	High bilirubin (conjugated) (> 5 µmol/L)	8 (16.3%, 7.3–30.9)	2 (5.6%, 0.7–18.7)	3 (30.0%, 6.7–65.3)
	Low albumin (< 35 g/L)	26 (53.1%, 39.3–66.6)	17 (47.2%, 30.4–64.5)	6 (60.0%, 26.2–87.8)
Lipid panels	High triglycerides (> 1.70 mmol/L)	0 (0.0%, 0.0–7.2)	7 (19.4%, 8.0–35.2)	0 (0.0%, 0.0–30.8)
	High total cholesterol (> 5.2 mmol/L)	2 (4.1%, 0.5–14.0)	8 (22.2%, 9.9–39.6)	0 (0.0%, 0.0–30.8)
	Low HDL (M:<1.45 mmol/L; F: <1.68 mmol/L)	48 (98.0%, 89.3–99.9)	31 (86.1%, 70.5–95.3)	8 (80.0%, 44.4–97.5)
	High LDL (> 3.0 mmol/L)	6 (12.2%, 4.6–24.9)	13 (36.1%, 20.8–53.8)	0 (0.0%, 0.0–30.8)
	Total cholesterol/HDL ratio (> 4.1)	26 (53.1%, 39.3–66.6)	16 (44.4%, 28.4–61.4)	4 (40.0%, 12.2–73.8)

Data are presented as counts and percentages of patients with biochemical abnormalities in respective cohorts at baseline; Percentages include 95% confidence intervals (CI) calculated by the normal approximation; Clinical cut-offs for biochemical abnormalities are according to standard definitions, Hypokalemia = serum potassium < 3.5 mmol/L; Hyperkalemia = serum potassium > 5.1 mmol/L; ALT = alanine aminotransferase; AST = aspartate aminotransferase; ALP = alkaline phosphatase; eGFR = estimated glomerular filtration rate.

**Table 5:** Serum biochemical abnormalities across cohorts at one month of anti-TB and/or diabetes treatment (Day 28, *N* = 88).

Parameter	Serum abnormalities	TB-Only (*n* = 47) Count (%, 95% CI)	TB-DMt (*n* = 33) Count (%, 95% CI)	TB-DMnt (*n* = 8) Count (%, 95% CI)
Serum Electrolyte	Hypokalemia (< 3.5 mmol/L)	1 (2.1%, 0.1–11.1)	3 (9.1%, 1.9–24.3)	3 (37.5%, 8.5–75.5)
	Hyperkalemia (> 5.1 mmol/L)	0 (0.0%, 0.0–7.5)	4 (12.1%, 3.3–29.7)	1 (12.5%, 0.3–52.7)
	Low sodium (< 136 mmol/L)	11 (23.4%, 12.5–37.2)	15 (45.5%, 28.1–63.9)	2 (25.0%, 3.2–65.1)
	Low chloride (< 98 mmol/L)	3 (6.4%, 1.3–17.5)	9 (27.3%, 13.2–45.9)	0 (0.0%, 0.0–35.4)
Renal panels	High urea (> 7.1 mmol/L)	0 (0.0%, 0.0–7.5)	1 (3.0%, 0.1–15.4)	0 (0.0%, 0.0–35.4)
	High creatinine (M: >106 µmol/L; F: >80 µmol/L)	0 (0.0%, 0.0–7.5)	4 (12.1%, 3.3–29.7)	2 (25.0%, 3.2–65.1)
	Low eGFR (18-41years < 89: >41 years < 60 mL/min/1.73m^2^)	3 (6.4%, 1.3–17.5)	4 (12.1%, 3.3–29.7)	1 (12.5%, 0.3–52.7)
Liver panels	High ALT (M: >41 IU/L; F: >33 IU/L)	5 (10.6%, 3.5–23.3)	5 (15.2%, 5.1–32.8)	1 (12.5%, 0.3–52.7)
	High AST (M: >40 IU/L; F: >32 IU/L)	7 (14.9%, 6.2–28.1)	5 (15.2%, 5.1–32.8)	1 (12.5%, 0.3–52.7)
	Low AST/ALT ratio (< 1)	5 (10.6%, 3.5–23.3)	8 (24.2%, 11.2–42.3)	0 (0.0%, 0.0–35.4)
	High AST/ALT ratio (> 2)	6 (12.8%, 4.8–26.9)	4 (12.1%, 3.3–29.7)	1 (12.5%, 0.3–52.7)
	High ALP (> 105 IU/L)	13 (27.7%, 15.5–42.4)	15 (45.5%, 28.1–63.9)	3 (37.5%, 8.5–75.5)
	High bilirubin (total) (> 20.5 µmol/L)	0 (0.0%, 0.0–7.5)	0 (0.0%, 0.0–10.6)	2 (25.0%, 3.2–65.1)
	High bilirubin (conjugated) (> 5 µmol/L)	2 (4.3%, 0.5–14.5)	5 (15.2%, 5.1–32.8)	4 (50.0%, 15.7–84.3)
	Low albumin (< 35 g/L)	15 (31.9%, 19.0–47.2)	11 (33.3%, 17.3–52.5)	4 (50.0%, 15.7–84.3)
Lipid panels	High triglycerides (> 1.70 mmol/L)	1 (2.1%, 0.1–11.1)	3 (9.1%, 1.9–24.3)	1 (12.5%, 0.3–52.7)
	High total cholesterol (> 5.2 mmol/L)	5 (10.6%, 3.5–23.3)	15 (45.5%, 28.1–63.9)	0 (0.0%, 0.0–35.4)
	Low HDL (M:<1.45 mmol/L; F: <1.68 mmol/L)	40 (85.1%, 71.4–93.9)	25 (75.8%, 57.9–89.0)	7 (87.5%, 47.3–99.7)
	High LDL (> 3.0 mmol/L)	10 (21.3%, 10.2–36.6)	14 (42.4%, 26.6–59.5)	0 (0.0%, 0.0–35.4)
	Total cholesterol/HDL ratio (> 4.1)	10 (21.3%, 10.2–36.6)	7 (21.2%, 9.1–39.6)	0 (0.0%, 0.0–35.4)

Data are presented as counts and percentages of patients with biochemical abnormalities in respective cohorts at day 28; Percentages include 95% confidence intervals (CI) calculated by the normal approximation; Clinical cut-offs for biochemical abnormalities are according to standard definitions, Hypokalemia = serum potassium < 3.5 mmol/L; Hyperkalemia = serum potassium > 5.1 mmol/L; ALT = alanine aminotransferase; AST = aspartate aminotransferase; ALP = alkaline phosphatase; eGFR = estimated glomerular filtration rate.

**Table 6:** Serum biochemical abnormalities across cohorts during anti-TB and diabetes treatment at two months (Day 56, *N* = 94).

Parameter	Serum abnormalities	TB-Only (*n* = 48) Count (%, 95% CI)	TB-DMt (*n* = 36) Count (%, 95% CI)	TB-DMnt (*n* = 10) Count (%, 95% CI)
Serum Electrolyte	Hypokalemia (< 3.5 mmol/L)	1 (2.1%, 0.1–11.3)	1 (2.8%, 0.1–14.5)	1 (10.0%, 0.3–44.5)
	Hyperkalemia (> 5.1 mmol/L)	1 (2.1%, 0.1–11.3)	1 (2.8%, 0.1–14.5)	0 (0.0%, 0.0–30.8)
	Low Sodium (< 136 mmol/L)	11 (22.9%, 12.6–37.4)	10 (27.8%, 14.2–45.2)	3 (30.0%, 6.7–65.3)
	Low chloride (< 98 mmol/L)	3 (6.3%, 1.3–17.6)	3 (8.3%, 1.8–22.6)	2 (20.0%, 2.5–55.6)
Renal panels	High urea (> 7.1 mmol/L)	0 (0.0%, 0.0–7.3)	1 (2.8%, 0.1–14.5)	0 (0.0%, 0.0–30.8)
	High creatinine (M: >106 µmol/L; F: >80 µmol/L)	2 (4.2%, 0.5–14.3)	2 (5.6%, 0.7–18.7)	1 (10.0%, 0.3–44.5)
	Low eGFR (18-41years < 89: >41 years < 60 mL/min/1.73m^2^)	2 (4.2%, 0.5–14.3)	4 (11.1%, 3.1–26.1)	1 (10.0%, 0.3–44.5)
Liver panels	High ALT (M: >41 IU/L; F: >33 IU/L)	5 (10.4%, 3.5–23.0)	4 (11.1%, 3.1–26.1)	1 (10.0%, 0.3–44.5)
	High AST (M: >40 IU/L; F: >32 IU/L)	8 (16.7%, 7.4–31.0)	5 (13.9%, 4.7–30.7)	1 (10.0%, 0.3–44.5)
	Low AST/ALT ratio (< 1)	6 (12.5%, 4.7–25.7)	9 (25.0%, 12.2–42.4)	0 (0.0%, 0.0–30.8)
	High AST/ALT ratio (> 2)	9 (18.8%, 9.0–32.0)	2 (5.6%, 0.7–18.7)	2 (20.0%, 2.5–55.6)
	High ALP (> 105 IU/L)	5 (10.4%, 3.5–23.0)	12 (33.3%, 19.6–49.6)	2 (20.0%, 2.5–55.6)
	High bilirubin (total) (> 20.5 µmol/L)	1 (2.1%, 0.1–11.3)	0 (0.0%, 0.0–7.2)	0 (0.0%, 0.0–30.8)
	High bilirubin (conjugated) (> 5 µmol/L)	2 (4.2%, 0.5–14.3)	2 (5.6%, 0.7–18.7)	1 (10.0%, 0.3–44.5)
	Low albumin (< 35 g/L)	12 (25.0%, 13.9–39.6)	9 (25.0%, 12.2–42.4)	6 (60.0%, 26.2–87.8)
Lipid panels	High triglycerides (> 1.70 mmol/L)	2 (4.2%, 0.5–14.3)	1 (2.8%, 0.1–14.5)	0 (0.0%, 0.0–30.8)
	High total cholesterol (> 5.2 mmol/L)	3 (6.3%, 1.3–17.6)	14 (38.9%, 23.1–56.5)	1 (10.0%, 0.3–44.5)
	Low HDL (M:<1.45 mmol/L; F: <1.68 mmol/L)	38 (79.2%, 64.7–89.2)	23 (63.9%, 46.2–79.2)	7 (70.0%, 34.8–93.3)
	High LDL (> 3.0 mmol/L)	9 (18.8%, 9.0–32.0)	17 (47.2%, 30.4–64.5)	1 (10.0%, 0.3–44.5)
	Total cholesterol/HDL ratio (> 4.1)	7 (14.6%, 6.0–27.6)	6 (16.7%, 6.4–34.5)	1 (10.0%, 0.3–44.5)

Data are presented as counts and percentages of patients with biochemical abnormalities in respective cohorts at day 56; Percentages include 95% confidence intervals (CI) calculated by the normal approximation; Clinical cut-offs for biochemical abnormalities are according to standard definitions, Hypokalemia = serum potassium < 3.5 mmol/L; Hyperkalemia = serum potassium > 5.1 mmol/L; ALT = alanine aminotransferase; AST = aspartate aminotransferase; ALP = alkaline phosphatase; eGFR = estimated glomerular filtration rate.

### Correlations among serum biochemical parameters

**Fig. 5:**
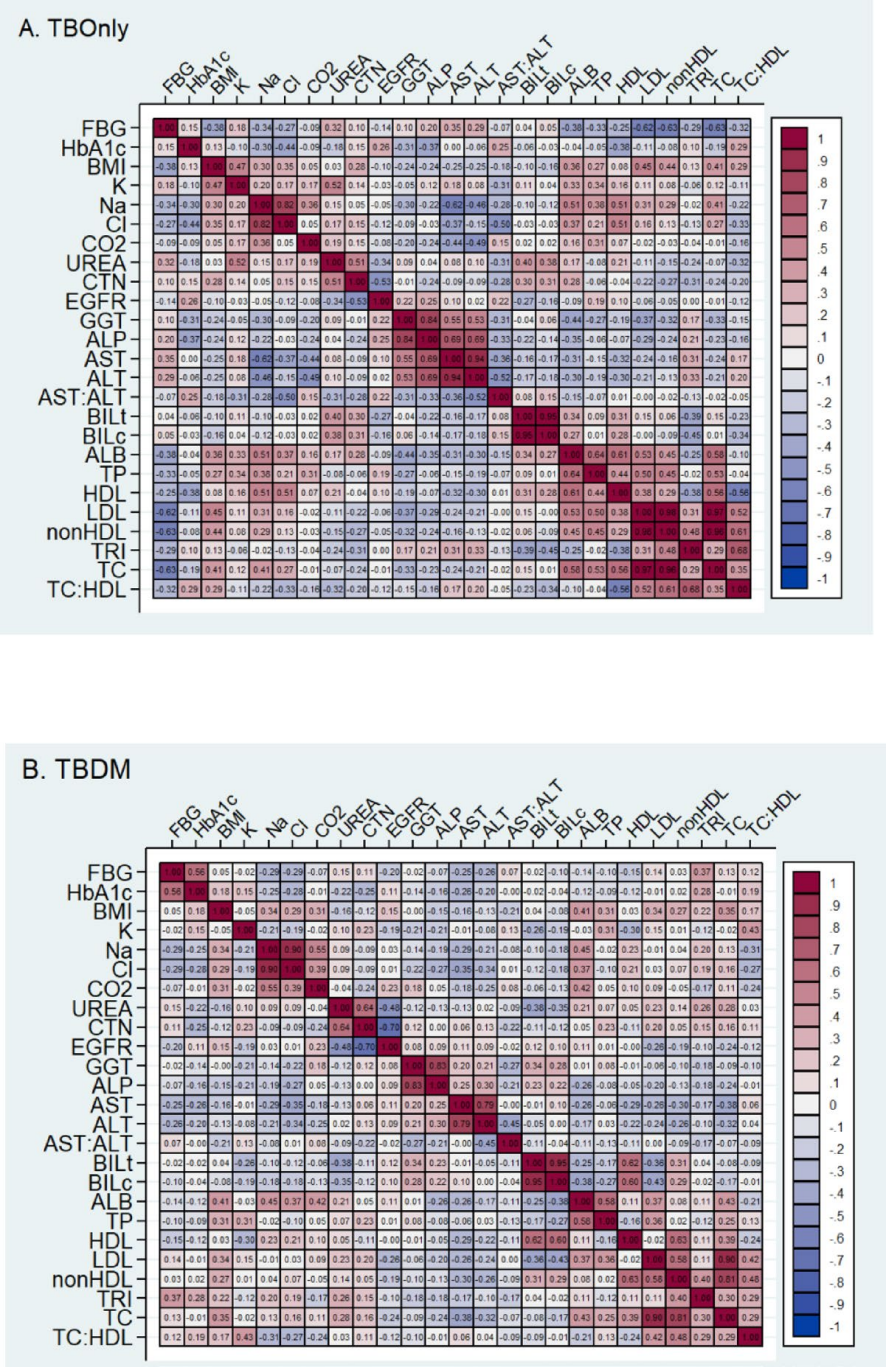
Correlation matrix of pre-therapeutic serum panels in patients with TB without (**A**) or with (**B**) DM. FBG: Fasting blood glucose, HbA1c: haemoglobin glycation, BMI: body mass index, TG: triglyceride, TC: total cholesterol, HDL: high-density lipoprotein cholesterol, LDL: low-density lipoprotein, Cl: chlorine, Na: sodium, K: potassium, CTN: creatinine, Bili: bilirubin, ALB: albumin, ALP: alanine phosphatase, GGT: γ-glutamyltransferase, ALT: Alanine aminotransferase, and AST: aspartate aminotransferase.

Among TB-DM patients, fasting blood glucose and HbA1c exhibited a moderate positive correlation (*r* = 0.56), whereas in TB-Only patients this association was weaker (*r* = 0.15) (Fig. 5). Albumin (ALB) showed moderate to strong correlations with HDL (*r* = 0.61), total cholesterol (*r* = 0.58), sodium (*r* = 0.51), and LDL (*r* = 0.53) in TB-Only patients. In TB-DM, however, these associations were substantially weakened, with albumin correlating only modestly to total cholesterol (*r* = 0.43) and sodium (*r* = 0.45). Both cohorts demonstrated strong positive correlations between ALT and AST (TB-Only: *r* = 0.94; TB-DM: *r* = 0.79), ALP and GGT (TB-Only: *r* = 0.84; TB-DM: *r* = 0.83), and creatinine and urea (TB-Only: *r* = 0.51; TB-DM: *r* = 0.64).

Notably, HbA1c in TB-DM patients showed weak negative correlations with HDL (*r* = − 0.12), urea (*r* = − 0.22), chloride (*r* = − 0.28), sodium (*r* = − 0.25), creatinine (*r* = − 0.25), ALT (*r* = − 0.20), and AST (*r* = − 0.26).

## Discussion

This study advances the understanding of comorbidities between tuberculosis (TB) and diabetes mellitus (DM) by profiling the clinical, metabolic, and biochemical parameters in patients throughout their treatment. Specifically, it reveals that TB-DM patients generally exhibit characteristics such as increased age, higher body mass indices, and a greater prevalence of hypertension. These factors may contribute to the increased susceptibility to TB through mechanisms of immunosenescence and metabolic syndrome, consistent with findings from existing epidemiological studies^11,12,17^.

Interestingly, significant biochemical abnormalities were noted in the TB-DM cohorts. In particular, the untreated subgroup (TB-DMnt) exhibited elevated liver enzymes, including bilirubin, gamma-glutamyl transferase (g-GT), and alkaline phosphatase (ALP) at baseline, indicative of hepatic stress or subclinical cholestasis likely associated with compounded effects of uncontrolled diabetes and concurrent TB infection. These results resonate with findings from prior studies suggesting that poor glycemic control in diabetic patients exacerbates complications during TB treatment^4,8^. Previous research has documented that variations in liver enzyme levels can complicate the initiation of therapies, such as Metformin^18,19^. Indeed, the heightened liver function tests (LFT) in this cohort may explain why clinicians might have withheld Metformin, fearing potential hepatotoxicity. Importantly, our data indicated that liver function markers normalized over time, suggesting that the initial hepatic impairment may have been transient, possibly due to the acute stresses of illness rather than an irreversible condition. The transient nature of these liver function test abnormalities in our study suggests that clinicians may consider re-evaluating the timing for Metformin initiation, ideally after a sufficient duration of TB treatment when LFTs stabilize. Future studies should aim to define clear timeframes for Metformin initiation, perhaps starting after two to four weeks of TB treatment, contingent upon the normalization of liver enzymes^19^.

Hyponatremia, defined as serum sodium levels less than 136 mmol/L, emerged as a significant finding within this cohort, particularly pronounced among TB-DMnt patients, where it affected 70% of the group. The elevated prevalence of hyponatremia suggests that untreated diabetes may exacerbate electrolyte disturbances as a result of increased inflammatory responses and metabolic dysregulation. The presence of hyponatremia can result from multiple factors, including the syndrome of inappropriate antidiuretic hormone secretion (SIADH), adrenal insufficiency, and nutritional deficiencies, all of which necessitate careful clinical management to avoid complications. Routine monitoring of serum sodium levels in TB patients, particularly in those with untreated diabetes, is critical to prevent adverse outcomes and ensure effective treatment.

Additionally, the finding of persistently higher glycated haemoglobin (HbA1c) levels in the TB-DM group supports the premise that TB exacerbates metabolic dysregulation, as TB infection has been shown to drive systemic inflammation that interferes with glucose homeostasis^17,20^. The pathogen *M. tuberculosis* induces immune-metabolic alterations, including the upregulation of inflammatory cytokines^6,17^, which not only impair macrophage function but also disrupt both glucose and lipid metabolism. Moreover, the pathogen may induce a Warburg-like effect, enhancing glycolysis and lactate production, which promotes systemic insulin resistance and contributes to hyperglycemia^17^.

Surprisingly, the TB-DM patients, particularly those treated with Metformin (TB-DMt); had a paradoxically healthier lipid profile at specific time points, characterized by higher HDL cholesterol than both TB-Only and TB-DMnt groups. Potential explanations for this include Metformin’s lipid-lowering properties, the impact of multidimensional factors including dietary habits, and genetic predispositions^12^. However, TB-DMnt patients consistently presented lower LDL levels as compared with the TB-DMt cohort, which may correlate with acute inflammatory responses suppressing LDL production or underlying nutritional deficiencies. Elevated triglycerides and total cholesterol levels across diabetic groups further enhance a pro-atherogenic state and suggest a critical link between dyslipidemia and increased cardiovascular disease risk in this population^12,17,21–23^.

The lipid profile observed may reflect intricate interactions between TB, diabetes, and lipid metabolism which may be influenced by immune-mediated modulation or alterations in gut microbiota^17,24^. It raises questions regarding whether higher HDL may serve as a compensatory mechanism in the face of insulin resistance or reflects lifestyle factors that favour less inflammatory states. Furthermore, the significantly increased levels of bilirubin, gamma-glutamyl transferase (g-GT) and alkaline phosphatase (ALP) observed in the TB-DMnt cohort, exceeding those of both the TB-Only and TB-DMt groups, may indicate heightened metabolic or treatment stress resulting from untreated diabetes^25^, or possible alcohol use (although baseline self-reported alcohol consumption did not significantly differ) or potentially lifestyle factors^8–10^. We acknowledge that our study lacked detailed behavioural data during anti-TB therapy, limiting our ability to draw definitive conclusions regarding lifestyle influences on hepatic function.

Contrary to expectation, renal function markers (urea, creatinine, eGFR) were not significantly worse in TB-DM or TB-DMnt patients at baseline or throughout follow-up. This may reflect the renal-protective effect of metformin among TB-DMt as well as possible survivour bias and rigorous exclusion criteria (excluding those with overt renal disease at baseline)^4,9,25,26^. This finding warrants further exploration to assess whether the initiation of Metformin correlates with improvements in renal function specifically within the TB-DM cohort.

Despite the absence of statistically significant differences in treatment-emergent adverse events (TEAEs) between patient groups, the increased incidence of TEAEs in TB-DM patients emphasizes the complexities involved in managing multiple comorbidities. Primary outcomes such as sputum culture conversion and treatment outcomes being similar regardless of DM status fortify evidence that effective TB care is attainable with comprehensive management^4^, though the metabolic perturbations identified necessitate integrated monitoring. Additionally, the prolonged production of blood-stained, mucopurulent sputum in the TB-DM cohorts indicated the extent of lung damage, suggesting a more severe presentation and poorer outcomes for individuals with DM^11^. Dyspnea was notably higher in TB-DM patients during the intensive phase, suggesting additional respiratory challenges in this group. These findings align with previous studies^7^, highlighting a consistent pattern in the coexistence of TB and DM.

The correlations observed among serum biochemical parameters were primarily expected, with significant associations between sodium and chloride levels reflecting their roles in maintaining osmotic balance. However, the weaker correlations noted in the TB-DM group, particularly between albumin and hepatic/lipid markers, imply that diabetes may disrupt established metabolic homeostasis^12,17,27^, potentially due to immune-metabolic crosstalk, cytokine effects, or treatment interactions. Such findings merit mechanistic exploration^28^.

This investigation stands out for its prospective design and detailed phenotyping, focusing on temporal biochemical changes. A key limitation is the unequal group sizes and potential for selection bias, particularly as the TB-DM group was older and included more hypertensive patients, both of which independently affect biochemical outcomes. The lack of antihypertensive medication data further complicates interpretation, as both aging and diuretic/exclusion therapies can influence sodium, chloride, and lipid profiles. The sample size, especially for the TB-DMnt group, restricted reliable estimation of certain indices and diagnostic power for less frequent events. Furthermore, the study did not address the potential for unmeasured confounders such as undiagnosed mild kidney disease or subclinical hepatic conditions.

Future studies should employ larger, matched cohorts, incorporate detailed antihypertensive treatment data, and stratify for age and comorbid risk. The interplay between anti-TB therapy timing, liver enzyme normalization, and diabetes management (metformin, insulin, or alternative agents) warrants focused clinical trials. It would also be valuable to prospectively monitor alcohol use and nutritional status as covariates with potential impact on liver and lipid outcomes.

In conclusion, this study highlights the intricate interplay between tuberculosis and diabetes mellitus, demonstrating specific metabolic and biochemical derangements associated with comorbidity. The findings emphasize the necessity of integrated management for these patients, including careful monitoring for hyponatremia and other metabolic imbalances, as well as the strategic timing of antidiabetic therapies. Continued investigations will enrich our understanding of TB-DM interactions and enhance clinical practices aimed at improving patient outcomes in this dual burden syndrome.

## Supplementary Information

Below is the link to the electronic supplementary material.

Supplementary Material 1

Supplementary Material 2

Supplementary Material 3

## Data Availability

All relevant data for arriving at the conclusions of this manuscript are located within the manuscript or as an attachment. Raw data can be obtained directly from the corresponding author at dyeboah-manu@noguchi.ug.edu.gh or through the NMIMR institutional data repository via PAsare@noguchi.ug.edu.gh.
