# Supplementary material for: Patients with tuberculosis and diabetes show altered clinical and biochemical parameters during anti-TB treatment
Source: Sci Rep. 2026 Feb 4;16:7266. doi: 10.1038/s41598-026-36529-8 (PMC12923770; doi:10.1038/s41598-026-36529-8)
Supplement: Supplementary file 1 — Supplementary Material 1 [file 41598_2026_36529_MOESM1_ESM.docx]

Supplementary table 1. Serum Biochemical Parameters at Baseline (t_0_)

|  |  |  |  | TB-DM subgroups | |  |  |  |  |
| --- | --- | --- | --- | --- | --- | --- | --- | --- | --- |
| Parameter  N=95 | Reference value | TB-Only  Median (IQR)  n=49 | TB-DM  Median (IQR)  n=46 | TB-DMt  Median (IQR)  n=36 | TB-DMnt  Median (IQR)  n=10 | TB-Only  Vs. TB-DM  P-Value | TB-Only  Vs. TB-DMt  P-Value | TB-Only  Vs. TB-DMnt  P-Value | TB-DMt  Vs. TB-DMnt  P-Value |
| Electrolyte |  |  |  |  |  |  |  |  |  |
| Potassium (K^+^) | 3.5–5.1 mmol/L | 4.3 (3.9–4.6) | 4.2 (4.0–4.7) | 4.4 (4.0–4.8) | 4.1 (3.9–4.4) | 0.626 | 0.346 | 0.363 | 0.166 |
| Sodium (Na^+^) | 136–145 mmol/L | 135 (134–137) | 135 (130–137) | 135 (132–137) | 132 (130–134) | 0.124 | 0.419 | 0.016* | 0.115 |
| Chloride (Cl^-^) | 98–107 mmol/L | 100 (98–103) | 98 (95–100) | 98 (96–100) | 95 (93–97) | 0.003** | 0.029* | 0.002** | 0.042 |
| Total HCO_3_^-^ (Bicarbonate) | 21–31 mmol/L | 26.2 (24.6–28.0) | 25 (24.0–27.1) | 25.5 (24.3–27.2) | 24.5 (23.6–25.9) | 0.348 | 0.624 | 0.136 | 0.236 |
| Renal panels |  |  |  |  |  |  |  |  |  |
| Urea | 2.1–7.1 mmol/L | 2.7 (2.3–3.2) | 3.1 (2.3–4.0) | 3.1 (2.3–4.0) | 2.8 (2.4–3.1) | 0.117 | 0.067 | 1.000 | 0.339 |
| Creatinine | 44–106 µmol/L | 62 (52–70) | 58 (51–65) | 57 (50–63) | 62 (58–73) | 0.240 | 0.110 | 0.581 | 0.126 |
| eGFR | >89 mL/min/1.73 m² | 89 (89–89) | 89 (89–89) | 89 (89–89) | 89 (89–89) | 0.158 | 0.170 | 0.358 | 0.977 |
| Liver function panels |  |  |  |  |  |  |  |  |  |
| Bilirubin (total) | 3.42–20.52 µmol/L | 9 (7–12) | 9 (7–13) | 9 (7–12) | 15.5 (11–19) | 0.404 | 0.904 | 0.007* | 0.005* |
| Bilirubin (conjugated) | <5 µmol/L | 2 (1–3) | 3 (2–4) | 2 (2–3) | 4 (3.5–6.5) | 0.252 | 0.844 | 0.005* | 0.002** |
| g-GT | <55 IU/L | 44 (28–72) | 62 (28–122) | 45 (27–92) | 137 (125–293) | 0.123 | 0.718 | 0.000*** | 0.001** |
| AST | 0–40 IU/L | 25 (21–31) | 26 (17–36) | 23 (17–34) | 65 (27–103) | 0.613 | 0.133 | 0.024* | 0.006* |
| ALT | 0–41 IU/L | 19 (13–27) | 20 (12–34) | 18.5 (12–28) | 47 (24–137) | 0.569 | 0.718 | 0.011* | 0.009* |
| ALP | 35–105 IU/L | 89 (77–122) | 108 (91–165) | 105 (91–134) | 170 (117–216) | 0.008* | 0.060 | 0.002** | 0.032* |
| Total Serum Protein | 64–83 g/L | 78 (75–81) | 74 (70–79) | 73 (69–80) | 77 (73–79) | 0.009* | 0.008* | 0.333 | 0.297 |
| Serum Albumin | 39.7–52 g/L | 34 (31–38) | 34 (30–37) | 35 (31–37) | 31 (28–35) | 0.571 | 0.919 | 0.167 | 0.188 |
| Lipid panels |  |  |  |  |  |  |  |  |  |
| Total Cholesterol (CHOL) | <5.2 mmol/L | 3.5 (2.9–4.1) | 4.0 (3.4–5.0) | 4.2 (3.6–5.1) | 3.2 (2.8–3.7) | 0.015* | 0.002** | 0.364 | 0.007* |
| LDL | <3.0 mmol/L | 2.2 (1.8–2.7) | 2.4 (1.9–3.0) | 2.6 (2.0–3.1) | 1.9 (1.6–2.2) | 0.370 | 0.087 | 0.098 | 0.023* |
| HDL | >1.45 mmol/L | 0.8 (0.7–1.0) | 1.0 (0.8–1.3) | 1 (0.8–1.4) | 0.9 (0.7–1.4) | 0.008* | 0.003* | 0.798 | 0.143 |
| Cholesterol / HDL Ratio | <4.1 | 4.2 (3.5–5.0) | 4.0 (3.4–4.9) | 4.0 (3.4–5.0) | 3.9 (3.4–4.6) | 0.496 | 0.653 | 0.395 | 0.630 |
| Triglycerides (TG) | <1.70 mmol/L | 0.8 (0.7–0.9) | 1.2 (1.0–1.4) | 1.2 (1.0–1.6) | 1.1 (0.9–1.3) | 0.000*** | 0.000*** | 0.025* | 0.329 |

P-values represent the results of pairwise comparisons between the three cohorts (TB-Only, TB-DMt, TB-DMnt) using two‐sample Wilcoxon rank‐sum (Mann–Whitney) test. Statistical significance is indicated as follows: *p < 0.05, **p < 0.01, ***p < 0.001. Data are presented as median (interquartile range).
