# Supplementary material for: Patients with tuberculosis and diabetes show altered clinical and biochemical parameters during anti-TB treatment
Source: Sci Rep. 2026 Feb 4;16:7266. doi: 10.1038/s41598-026-36529-8 (PMC12923770; doi:10.1038/s41598-026-36529-8)
Supplement: Supplementary file 2 — Supplementary Material 2 [file 41598_2026_36529_MOESM2_ESM.docx]

Supplementary table 2. Serum Biochemical Parameters at Follow-up Day 28 (t_28_)

|  |  |  |  | TB-DM subgroups | |  |  |  |  |
| --- | --- | --- | --- | --- | --- | --- | --- | --- | --- |
| Parameter  N=90 | Reference value | TB-Only  Median (IQR)  n=47 | TB-DM  Median (IQR)  n=43 | TB-DMt  Median (IQR)  n=35 | TB-DMnt  Median (IQR)  n=8 | TB-Only  Vs. TB-DM  P-Value | TB-Only  Vs. TB-DMt  P-Value | TB-Only  Vs. TB-DMnt  P-Value | TB-DMt  Vs. TB-DMnt  P-Value |
| Electrolyte |  |  |  |  |  |  |  |  |  |
| Potassium (K) | 3.5–5.1 mmol/L | 4.0 (3.7–4.2) | 4.1 (3.8–4.5) | 4.1 (3.8–4.5) | 3.9 (3.4–4.3) | 0.325 | 0.157 | 0.487 | 0.228 |
| Sodium (Na) | 136–145 mmol/L | 137 (136–138) | 136 (134–138) | 136 (134–138) | 138 (136–139) | 0.216 | 0.078 | 0.400 | 0.181 |
| Chloride (Cl) | 98–107 mmol/L | 103 (102–105) | 99 (98–102) | 99 (97–102) | 102 (102–105) | 0.000*** | 0.000*** | 0.952 | 0.012* |
| Total HCO3- (Bicarbonate) | 21–31 mmol/L | 26.5 (24.3–28.1) | 26.8 (25.0–29.0) | 26.9 (26.0–29.0) | 25.1 (24.1–27.0) | 0.538 | 0.288 | 0.390 | 0.128 |
| Renal panels |  |  |  |  |  |  |  |  |  |
| Urea | 2.1–7.1 mmol/L | 2.4 (2.0–2.7) | 2.4 (2.0–3.6) | 2.4 (2.0–3.7) | 2.2 (2.1–2.9) | 0.153 | 0.091 | 0.933 | 0.453 |
| Creatinine | 44–106 µmol/L | 63 (51–70) | 58 (47–65) | 58 (47–64) | 58 (54–66) | 0.088 | 0.069 | 0.685 | 0.553 |
| eGFR | >89 mL/min/1.73 m² | 89 (89–89) | 89 (89–89) | 89 (89–89) | 89 (88–89) | 0.041* | 0.019* | 0.680 | 0.214 |
| Liver function panels |  |  |  |  |  |  |  |  |  |
| Bilirubin (total) | 3.42–20.52 µmol/L | 9 (7–11) | 9 (8–13) | 9 (8–12) | 15 (9–20) | 0.242 | 0.627 | 0.030 | 0.053 |
| Bilirubin (conjugated) | <5 µmol/L | 3 (2–3) | 3 (2–4) | 3 (2–4) | 5 (3–8) | 0.380 | 0.932 | 0.016* | 0.032* |
| g-GT | <55 IU/L | 49 (37–69) | 67 (38–106) | 57 (36–93) | 96 (43–152) | 0.218 | 0.399 | 0.134 | 0.190 |
| AST | 0–40 IU/L | 27 (22–33) | 25 (21–31) | 23 (21–31) | 27 (23–37) | 0.170 | 0.096 | 0.864 | 0.310 |
| ALT | 0–41 IU/L | 16 (14–28) | 19 (13–26) | 19 (13–26) | 18 (14–20) | 0.834 | 0.745 | 0.836 | 0.673 |
| ALP | 35–105 IU/L | 92 (70–108) | 101 (82–122) | 101 (83–122) | 95 (75–118) | 0.**055** | 0.039* | 0.670 | 0.454 |
| Total Serum Protein | 64–83 g/L | 77 (74–81) | 73 (70–77) | 72 (68–77) | 75 (73–79) | 0.001** | 0.000*** | 0.435 | 0.111 |
| Serum Albumin | 39.7–52 g/L | 37 (34–40) | 36 (32–39) | 36 (33–39) | 34 (29–37) | 0.261 | 0.544 | 0.077 | 0.154 |
| Lipid panels |  |  |  |  |  |  |  |  |  |
| Total Cholesterol (CHOL) | <5.2 mmol/L | 4.1 (3.5–4.6) | 4.3 (3.7–5.5) | 4.7 (3.7–5.6) | 4.0 (3.7–4.3) | 0.069 | 0.018* | 0.519 | 0.098 |
| LDL | <3.0 mmol/L | 2.5 (2.0–2.9) | 2.4 (2.2–3.1) | 2.6 (2.2–3.3) | 2.3 (1.9–2.4) | 0.484 | 0.154 | 0.117 | 0.047* |
| HDL | >1.45 mmol/L | 1.2 (1.0–1.4) | 1.3 (1.0–1.7) | 1.3 (1.0–1.7) | 1.3 (1.1–1.4) | 0.156 | 0.133 | 0.718 | 0.415 |
| Cholesterol / HDL Ratio | <4.1 | 3.3 (2.9–3.9) | 3.4 (2.7–3.9) | 3.4 (2.7–3.9) | 3.3 (3.0–3.7) | 0.894 | 0.785 | 0.765 | 0.684 |
| Triglycerides (TG) | <1.70 mmol/L | 0.8 (0.6–1.0) | 1.1 (0.9–1.4) | 1.1 (0.9–1.4) | 1.2 (0.8–1.4) | 0.000*** | 0.001** | 0.040* | 0.731 |

P-values represent the results of pairwise comparisons between the three cohorts (TB-Only, TB-DMt, TB-DMnt) using two‐sample Wilcoxon rank‐sum (Mann–Whitney) test. Statistical significance is indicated as follows: *p < 0.05, **p < 0.01, ***p < 0.001. Data are presented as median (interquartile range).
