# Supplementary material for: Patients with tuberculosis and diabetes show altered clinical and biochemical parameters during anti-TB treatment
Source: Sci Rep. 2026 Feb 4;16:7266. doi: 10.1038/s41598-026-36529-8 (PMC12923770; doi:10.1038/s41598-026-36529-8)
Supplement: Supplementary file 3 — Supplementary Material 3 [file 41598_2026_36529_MOESM3_ESM.docx]

Supplementary table 3. Serum Biochemical Parameters at Follow-up Day 56 (t_56_)

|  |  |  |  | TB-DM subgroups | |  |  |  |  |
| --- | --- | --- | --- | --- | --- | --- | --- | --- | --- |
| Parameter  N=94 | Reference value | TB-Only  Median (IQR)  n=48 | TB-DM  Median (IQR)  n=46 | TB-DMt  Median (IQR)  n=36 | TB-DMnt  Median (IQR)  n=10 | TB-Only  Vs. TB-DM  P-Value | TB-Only  Vs. TB-DMt  P-Value | TB-Only  Vs. TB-DMnt  P-Value | TB-DMt  Vs. TB-DMnt  P-Value |
| Electrolyte |  |  |  |  |  |  |  |  |  |
| Potassium (K) | 3.5–5.1 mmol/L | 3.9 (3.8–4.3) | 4.2 (4.0–4.4) | 4.2 (3.9–4.4) | 4.3 (4.1–4.4) | 0.066 | 0.135 | 0.109 | 0.469 |
| Sodium (Na) | 136–145 mmol/L | 137(136–138) | 138(135–139) | 138 (135–139) | 138 (135–138) | 0.439 | 0.364 | 0.967 | 0.610 |
| Chloride (Cl) | 98–107 mmol/L | 104 (102–105) | 101 (100–103) | 101 (100–103) | 101 (98–105) | 0.005* | 0.006* | 0.149 | 0.717 |
| Total HCO3- (Bicarbonate) | 21–31 mmol/L | 26.2 (24.6–28.0) | 26.1 (24.1–27.8) | 26.3 (23.9–27.8) | 26.0 (25.3–28.3) | 0.802 | 0.816 | 0.879 | 0.899 |
| Renal panels |  |  |  |  |  |  |  |  |  |
| Urea | 2.1–7.1 mmol/L | 2.4 (2.0–3.1) | 2.8 (2.2–3.5) | 2.7 (1.9–3.6) | 2.9 (2.4–3.4) | 0.273 | 0.560 | 0.099 | 0.514 |
| Creatinine | 44–106 µmol/L | 59 (51–70) | 54 (44–60) | 50 (42–59) | 58 (54–65) | 0.020* | 0.007* | 0.880 | 0.119 |
| eGFR | >89 mL/min/1.73 m² | 89 (89–89) | 89 (89–89) | 89 (89–89) | 89 (89–89) | 0.930 | 0.931 | 0.677 | 0.663 |
| Liver function panels |  |  |  |  |  |  |  |  |  |
| Bilirubin (total) | 3.42–20.52 µmol/L | 8 (7–11) | 8 (7–10) | 7 (6–9) | 9 (8–13) | 0.485 | 0.149 | 0.170 | 0.026* |
| Bilirubin (conjugated) | <5 µmol/L | 2 (2–3) | 2 (1–3) | 2 (1–3) | 3 (2–3) | 0.731 | 0.458 | 0.449 | 0.286 |
| g-GT | <55 IU/L | 38 (32–55) | 62 (38–98) | 60 (38–93) | 91 (41–154) | 0.008* | 0.024* | 0.032* | 0.305 |
| AST | 0–40 IU/L | 28 (24–33) | 25 (20–31) | 24 (20–29) | 27 (18–39) | 0.029* | 0.019* | 0.544 | 0.729 |
| ALT | 0–41 IU/L | 18 (14–22) | 18 (13–27) | 20 (13–28) | 16 (12–20) | 0.864 | 0.566 | 0.401 | 0.323 |
| ALP | 35–105 IU/L | 85 (72–95) | 89 (81–111) | 90 (82–114) | 87 (71–105) | 0.047* | 0.030* | 0.642 | 0.613 |
| Total Serum Protein | 64–83 g/L | 75 (72–78) | 74 (70–78) | 74 (69–78) | 76 (73–79) | 0.679 | 0.356 | 0.331 | 0.174 |
| Serum Albumin | 39.7–52 g/L | 37 (35–39) | 37 (34–40) | 38 (35–41) | 34 (30–37) | 0.802 | 0.556 | 0.043* | 0.040* |
| Lipid panels |  |  |  |  |  |  |  |  |  |
| Total Cholesterol (CHOL) | <5.2 mmol/L | 4.0 (3.5–4.6) | 4.5 (3.6–5.5) | 4.8 (3.9–5.9) | 3.8 (3.3–4.4) | 0.030* | 0.004** | 0.479 | 0.038* |
| LDL | <3.0 mmol/L | 2.4 (2.0–2.7) | 2.7 (2.1–3.4) | 2.9 (2.2–3.5) | 2.2 (1.8–2.4) | 0.065 | 0.007* | 0.274 | 0.026* |
| HDL | >1.45 mmol/L | 1.2 (1.1–1.5) | 1.5 (1.2–1.6) | 1.5 (1.2–1.7) | 1.3 (1.0–1.6) | 0.057 | 0.032* | 0.768 | 0.377 |
| Cholesterol / HDL Ratio | <4.1 | 3.2 (2.9–3.8) | 3.3 (3.0–3.7) | 3.3 (3.–3.8) | 3.2 (2.9–3.4) | 0.726 | 0.536 | 0.649 | 0.330 |
| Triglycerides (TG) | <1.70 mmol/L | 0.8 (0.6–0.9) | 1.0 (0.8–1.2) | 1.0 (0.8–1.2) | 1.0 (0.7–1.0) | 0.008* | 0.017* | 0.071 | 0.922 |

P-values represent the results of pairwise comparisons between the three cohorts (TB-Only, TB-DMt, TB-DMnt) using two‐sample Wilcoxon rank‐sum (Mann–Whitney) test. Statistical significance is indicated as follows: *p < 0.05, **p < 0.01, ***p < 0.001. Data are presented as median (interquartile range).
